# Friend or Foe? Revising the Role of Oxygen in the Tribological Performance of Solid Lubricant MoS_2_

**DOI:** 10.1021/acsami.2c15706

**Published:** 2022-12-05

**Authors:** Andrey Bondarev, Ilia Ponomarev, Ruslan Muydinov, Tomas Polcar

**Affiliations:** † Department of Control Engineering, Faculty of Electrical Engineering, Czech Technical University in Prague, Technicka 2, 16627 Prague 6, Czech Republic; ‡ Institute of High-Frequency and Semiconductor System Technologies, Technical University Berlin, Einsteinufer 25, 10587 Berlin, Germany

**Keywords:** MoS_2_, solid lubricant, tribology, microstructure, ReaxFF

## Abstract

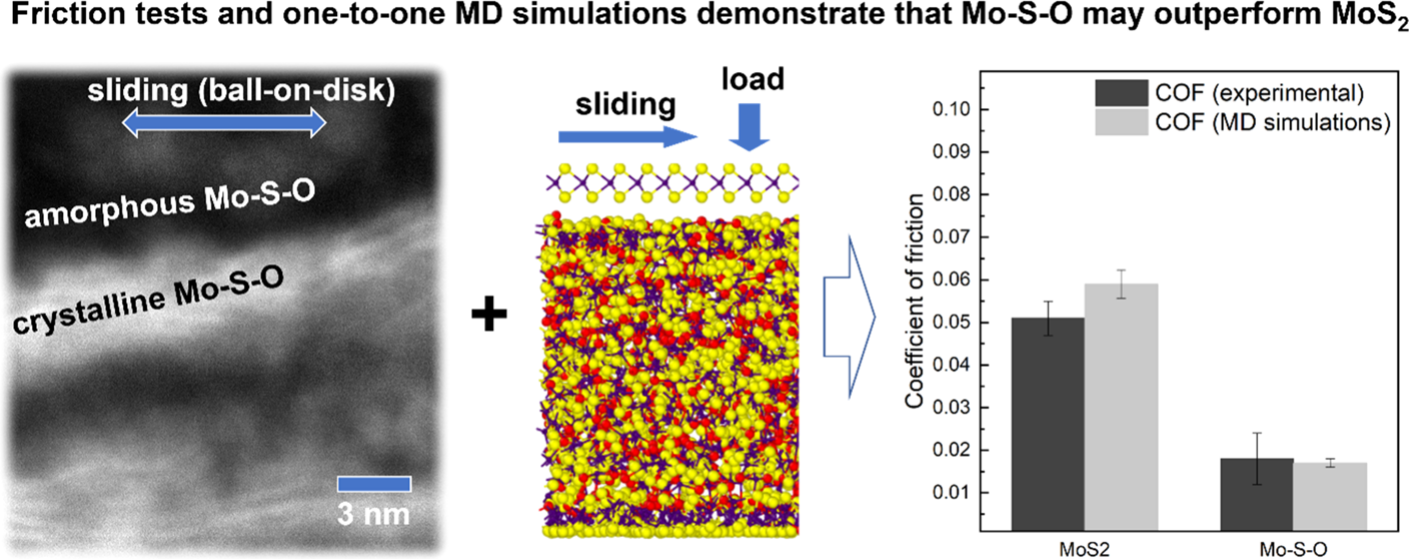

Molybdenum disulfide (MoS_2_) is a solid lubricant used in various forms, such as a dry lubricant by itself or as a component of a more complex coating. In both these forms, the effect of oxygen contamination on the sliding properties of the MoS_2_ coatings is traditionally considered detrimental, resulting in expensive technological processes to produce pure MoS_2_. Here, it is shown that the high oxygen content does not necessarily hinder the solid lubricant properties and may even result in a lower friction and wear when compared to pure MoS_2_. Mo–S–O coatings were fabricated by unbalanced magnetron sputtering and tribologically tested under vacuum conditions. Oxygen caused amorphization of the as-deposited coatings but did not prevent the triboactivated formation of an ultra-thin crystalline MoS_2_ tribolayer with the incorporated oxygen. Such an imperfect tribolayer was found to reduce the coefficient of friction to 0.02, a value lower than that of pure MoS_2_. Moreover, owing to the higher density and hardness of oxygen-containing films, the wear rate was also found to be lower. Molecular dynamics simulations performed using a newly developed Mo–S–O force field confirmed that such an imperfect tribolayer can mitigate friction in a manner comparable to that of MoS_2_.

## 1 Introduction

Transition-metal dichalcogenides (TMDs) are intrinsic solid lubricants exhibiting a layered hexagonal structure and a weak van der Waals bonding between basal planes in the crystallographic lattice,^1^ which provide low shear stress values under sliding and thus a low coefficient of friction (COF).^2^ TMDs, in both the 2D^3,4^ and coating forms, are the most popular materials used in tribological applications. They are exceptional solid lubricants under vacuum or a protective atmosphere, but their tribological properties rapidly deteriorate in dry and, particularly, humid air. Although many TMDs exhibit very similar tribological properties (e.g., WS_2_, WSe_2_, and MoSe_2_),^5−7^ molybdenum disulfide (MoS_2_) dominates the market.

The effect of oxygen on the tribological properties of TMDs is normally considered detrimental. The interaction of the film with oxygen can be split into three phases. First, during magnetron sputtering, oxygen is incorporated into the growing film from the residual atmosphere.^8^ Then, storing the film in the open air can cause oxidation.^9,10^ Finally, the coatings experience a negative influence of the molecular or atomic oxygen from the surrounding atmosphere on their tribological properties.^11−15^ Despite the origin, formation of a noticeable amount of molybdenum oxides on the sliding surface has been reported as the reason behind the poorer tribological properties.^2,16−18^

To enhance their tribological and mechanical properties, TMDs have been fabricated with a dense^19^ or gradient structure^6^ or combined with different additives to obtain a composite structure, including metallic (Ti,^20−22^ Zr,^23^ Cr,^22^ Au,^24,25^ Ni,^26^ Fe,^26^ Cu,^27^ Ag,^28^ Nb,^29^ and Pb^20,30^) and non-metallic elements (C,^31−33^ N,^34^ and F^35^). Further research investigated multicomponent systems containing TMDs;^36,37^ in extreme cases, there are five dopants.^38^ However, such complex systems are often difficult to upscale for industrial production; moreover, the detailed investigation of friction and wear mechanisms and their simulation at an atomic scale are almost impossible.

With certainty, one can say that the performance of TMDs is enhanced by doping, but what are the reasons for that? The enhancement of hardness, Young’s modulus, roughness, and density of the MoS_2_ coatings with alloying are responsible for the high load-bearing capacity and improved friction performance. Also, sensitivity to the environment can be reduced by using sacrificial dopants known as “oxygen getters”.^7,20,22,23,39^ In any scenario, oxygen in all forms has been labeled as the “foe” and something undesirable for MoS_2_-based materials.

To reduce the oxygen content, technologically complex and expensive processes are used, such as ultra-high vacuum deposition^40^ and storage in a specialized protective environment.^41^ The tribological applications are thus often limited to a protective atmosphere (e.g., dry nitrogen) or vacuum. However, the literature describing the tribological properties of MoS_2_ often neglects oxygen embedded into the coating during deposition. In fact, as-deposited coatings with the oxygen content as high as 12 at. % are labeled as “pure” MoS_2_,^19,20,23,30,42−46^ which may contribute to the contradictive data about the mechanical and tribological properties of the MoS_2_ coatings. For example, the hardness values from 0.13 to 7.1 GPa are reported for MoS_2_ coatings;^23,30,42−48^ such a difference can hardly be explained just by the dissimilarities in the porosity, structure, or grain size. At the same time, in an early work from Fleischauer, the highest COF values were found for the Mo–S–O coatings with a concentration of O between 1 and 10 at. %, which is the most common range of O contamination originating from the deposition processes.^49^ Adsorbed oxygen on the surface of the deposited MoS_2_ also plays a negative role during the first friction cycles, while after a running-in period, the COF becomes similar, irrespective of the amount of the oxygen in the topmost layers.^50^

The low friction stems from the formation of a thin nanometer-scale tribolayer consisting of a few MoS_2_ layers at the coating interface.^51−53^ Such a tribolayer is the result of a complex mechano-chemical process driven by high temperature in the contact, high contact pressure, and shear stress. Nanoscale phenomena also include the effects of thickness and orientation on the friction performance of the MoS_2_ layers.^54−56^

A reduction in the performance of the MoS_2_ coatings may be caused by the ambient oxygen, surface oxygen adsorbed during storage, and atomic oxygen in the outer space; these phenomena are widely discussed in the literature. That said, missing data on the exact amount of oxygen present in the MoS_2_-based materials and its impact on the structure–properties–performance triangle handicap a full comprehension of the subject.

To pinpoint the effects of oxygen on the performance of MoS_2_ coatings, we have prepared a series of O-doped (intentionally “contaminated”) MoS_2_ coatings and analyzed their frictional behavior under vacuum conditions. A combination of multi-scale analyses of the sliding interface, together with molecular dynamics (MD) simulations enabled by newly developed Mo–S–O force fields, sheds light on oxygen’s role in sliding and offers an alternative take to the traditional view of its detrimental effect.

## 2 Experimental and Computational Details

### 2.1 Deposition of the Coatings

The MoS_2_ and Mo–S–O coatings were deposited by unbalanced DC magnetron sputtering at a total gas pressure of 0.5 Pa in Ar, Ar + 5% O_2_, and Ar + 10% O_2_ using an AJA Orion 4 machine (AJA International). The coatings were deposited using a MoS_2_ disc target (51 mm in diameter). A Cr target was used for the deposition of a thin interlayer between the substrate and the functional coating. The Cr interlayer deposition (Cr target power of 200 W) was performed prior to MoS_2_ for 15 min and during the first 2 min of the process. A power of 80 W was applied for the deposition of the MoS_2_ and Mo–S–O coatings, and the deposition time was 210 min. During the deposition, no additional substrate heating was used. A bias voltage of 50 V was applied during the whole deposition process. The substrates (Si wafers and AISI D2 steel disks) were ultrasonically cleaned in isopropyl alcohol for 15 min and then plasma-cleaned in a vacuum chamber using an RF plasma source for 15 min prior to deposition.

### 2.2 Microstructural Characterization

The microstructure of the coatings was examined by scanning electron microscopy (SEM) using a Verios 460L (FEI) and MIRA3 (TESCAN) microscope and transmission electron microscopy (TEM) using a Tecnai TF20 X-Twin microscope (FEI) and Titan^3^ Themis 60-300 (FEI) with a Super-X EDS detector (Thermo Fisher Scientific). A dual-beam Helios NanoLab 660 microscope (FEI) was employed for the fabrication of lamellae from the wear track areas and the contact area on the counterpart surface; the lamellae were mounted on an FIB lift-out grid. X-ray photoelectron spectroscopy (XPS) analyses were performed using a Kratos XPS spectrometer (Shimadzu) equipped with a monochromated Al Kα X-ray source (*h*ν = 1486.6 eV). During the measurements, the base pressure inside the XPS chamber was kept constant at around 5 × 10^–9^ Torr. High-resolution XPS spectra were recorded with a 0.1 eV step. Surface etching was performed using a gas cluster ion source (GCIS) in a 20 kV Ar500^+^ cluster mode; the etching crater size was 1.9 mm × 1 mm, and the etching time was 600 s. The obtained XPS spectra were further calibrated by shifting the binding energy (BE) scale to center the adventitious C 1s spectral component (C–C, C–H) at 284.8 eV. Raman spectroscopy studies were done using an Xplora device with a laser wavelength λ = 532 nm (HORIBA Scientific). X-ray diffraction (XRD) measurements were performed using a Bruker D8 Discover system with Cu Kα radiation. The incident angle was 1°, with 2θ steps of 0.03° and an acquisition time of 5 s per step. From the primary side, an X-ray mirror and a 0.2 mm slit were used. The secondary side included the 0.25° equatorial soller and LYNXEYE XE-T detector in a 1D open mode. The sample surface was set up via repeated *z*-/ω-scans parallel to the beam at 2θ = 0°. The coatings were characterized in terms of their hardness, Young’s modulus, and elastic recovery using a TI 950 (Hysitron) nanoindenter equipped with a Berkovich diamond tip. The measurements were carried out using the depth control mode; for all the coatings, the maximal penetration depth was 100 nm (<10% of thickness) to minimize the substrate effect. The hardness and effective Young’s modulus were calculated using the Pharr and Oliver method with a calibration on fused silica. A custom-made vacuum pin-on-disk tribometer (Czech Technical University in Prague) was used to measure the tribological characteristics of the coatings. The samples were tested using the following conditions: counterpart—100Cr6 ball (6 mm diameter), linear speed—5 cm/s, normal load—5 N, and 5000 cycles (∼190 m). Tribological tests were carried out in a moderate vacuum (∼1 × 10^–3^ Pa) environment. The wear track profiles were acquired using a 3D optical profilometer Zygo NV7200 (Zygo).

### 2.3 MD Simulations

In the MD simulations, we used the ReaxFF^57^ force field as implemented in LAMMPS^58^ (reax/c package^59^). Details of the ReaxFF parameter set we applied in the study are given in the Supporting Information (Figures S8 and S9). In our simulations, we used a time step of 0.5 fs.

To model the Mo–S–O structure, we placed 560 Mo atoms, 880 S atoms, and 360 O atoms (MoS_2_ + MoO_3_ stoichiometry, 20 at. % O) randomly at slightly displaced orthogonal sites of a cell with dimensions of 27.791 × 28.881 × 38.000 Å. The cell was periodic in *x*- and *y*-directions, with Lennard-Jones walls on the edges of the cell in the *z*-direction. The system was further annealed at 2000 K: we heated it to 2000 K within 200 ps, held it at 2000 K for 1 ns, and cooled it to 0 K within 200 ps.

In all the sliding simulations, the outer layers of the S (and O, in the case of Mo–S–O) and Mo atoms on the top and the bottom of the models were treated as rigid bodies. The bottom rigid body was fixed, while the top one was forced to move along the *x*-direction with a constant velocity of 10 m/s. Extra forces corresponding to a normal load of 0.5, 1, 1.5, and 2 GPa were applied to the top rigid layer. The temperature of the system was 1500 K. For each setup (model, normal load), we performed three independent runs. The sliding stage of the simulation lasted for 2 ns. Throughout each simulation, the forces acting on the top body in the *x*- and *z*-directions and averages of those forces were recorded, which allowed us to find the friction coefficients as the slopes of the friction force versus the normal load relation.

## 3 Results and Discussion

**Figure 1:**
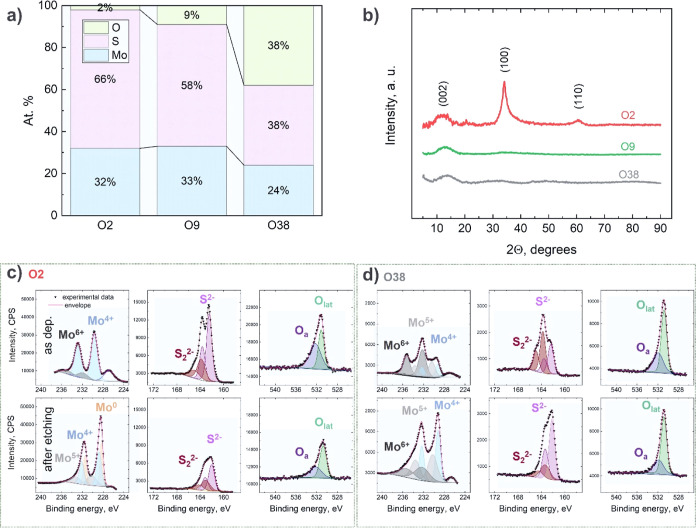
(a) Chemical composition and (b) GIXRD patterns of the O2, O9, and O38 coatings. High-resolution XPS Mo 3d, S 2p, and O 1s spectra of (c) O2 and (d) O38 as-deposited coatings before and after etching.

Figure 1a shows the chemical compositions of the coatings deposited in Ar, Ar + 5% O_2_, and Ar + 10% O_2_ atmospheres (hereafter denoted as O2, O9, and O38) determined by the EDX from the cross sections of the coatings in order to minimize the errors caused by the surface contamination. The O2 coating deposited in Ar contains 2 at. % oxygen from the residual atmosphere in the chamber. The ratio between Mo and S in the coatings deposited in pure Ar is very close to the MoS_2_ stoichiometry. A higher O flow into the deposition chamber leads to an expected increase in the O concentration; the oxygen preferentially displaces sulfur in the coating composition, while the molybdenum concentration is changed only slightly.

Figure 1b shows the grazing incidence XRD (GIXRD) patterns of all the coatings. For O2, the characteristic peak at 34° and small features at 14 and 60° 2θ correspond to the (100), (002), and (110) planes of h-MoS_2_ (JCPDS-37-1492), respectively; the pattern and peak broadness suggest a poorly crystalline structure of MoS_2_.^30,60^ The O9 and O38 coatings are X-ray amorphous, which is common for alloyed MoS_2_ coatings.^20,39,44^ XPS analyses provided a deeper insight into the chemical states of Mo and S of the pristine surface, and after the etching by GCIS, Mo 3d, S 2p, and O 1s XPS high-resolution spectra were analyzed. The deconvoluted Mo 3d XPS spectrum (Figure 1c) shows two 3d_5/2_ and 3d_3/2_ doublets indicative of Mo^4+^ (blue-colored doublet) and Mo^6+^ (gray-colored doublet) species, respectively. The dominant Mo^4+^ doublet corresponds to MoS_2_, and the Mo^6+^ doublet suggests the presence of MoO_3_, the origin of which is probably due to the exposure to the ambient atmosphere after deposition. Also, the S 2s peak at 226.9 eV BE is clearly seen (Figure 1c). The S 2p spectrum can be deconvoluted into two doublets assigned to S^2–^ in MoS_2_ (magenta-colored) and S_2_^2–^ species (dark red-colored). A sulfur doublet with a high BE can be associated with bridging S_2_^2–^, shared S_2_^2–^, and/or related to the MoO_*x*_S_*y*_ oxysulfide compounds.^61−66^ Since the Mo 3d spectrum of the O2 coating before etching shows no feature assigned to MoO_*x*_S_*y*_, the S_2_^2–^ species may be attributed to the bridging S_2_^2–^ and shared S_2_^2–^ in MoS_*x*_.^65−67^ The O 1s spectrum can be deconvoluted into two peaks at 532.0 and 530.8 eV, which are ascribed to the adsorbed oxygen and lattice oxygen, respectively.^63,68^ The spectrum after etching of the surface shows the change of the chemical states of Mo; the doublets belonging to Mo^0^ (metallic Mo), Mo^4+^ (MoS_2_), and Mo^5+^ (MoO_*x*_S_*y*_) contribute to the lineshape. The S 2p spectrum consists of the same S^2–^ and S_2_^2–^ species, but the intensity decreases significantly. This intensity decrease of the S 2p spectrum, together with the appearance of a zero-valent Mo, indicates a partial decomposition of MoS_2_ and a loss of sulfur caused by Ar^+^ bombardment.^69^ The low-intensity Mo^5+^ doublet assigned to MoO_*x*_S_*y*_ confirms the presence of oxygen in the MoS_2_ coatings from the residual atmosphere during deposition. The spectrum weight of the O 1s component assigned to the absorbed oxygen species is noticeably decreased after etching. The Mo 3d and S 2p spectra obtained for the O38 coating (Figure 1d) show the presence of MoS_2_, MoO_*x*_S_*y*_, and MoO_3_ compounds, either before or after etching. The doublets assigned to the O-containing compounds (MoO_*x*_S_*y*_ and MoO_3_) dominate the Mo 3d spectrum before etching, and after removal of the surface layer, the MoS_2_ feature becomes well pronounced. The fitted O 1s spectrum obtained from the uppermost surface of O38 and after etching demonstrates the same features as those for the O2 coating. The spectra from the whole batch of the coatings are shown in Figure S1. In summary, XPS provides several important indications: (1) the surface of the MoS_2_ (O2) and Mo–S–O (O9, O38) coatings contains more oxygen, probably because of adsorption, but below the surface, MoS_2_ (Mo^4+^), MoO_*x*_S_*y*_ (Mo^5+^), and MoO_3_ (Mo^6+^) coexist even in the case of the low concentration of S in the coating composition; (2) O-doped coatings are less sensitive to etching by Ar^+^ clusters.

**Figure 2:**
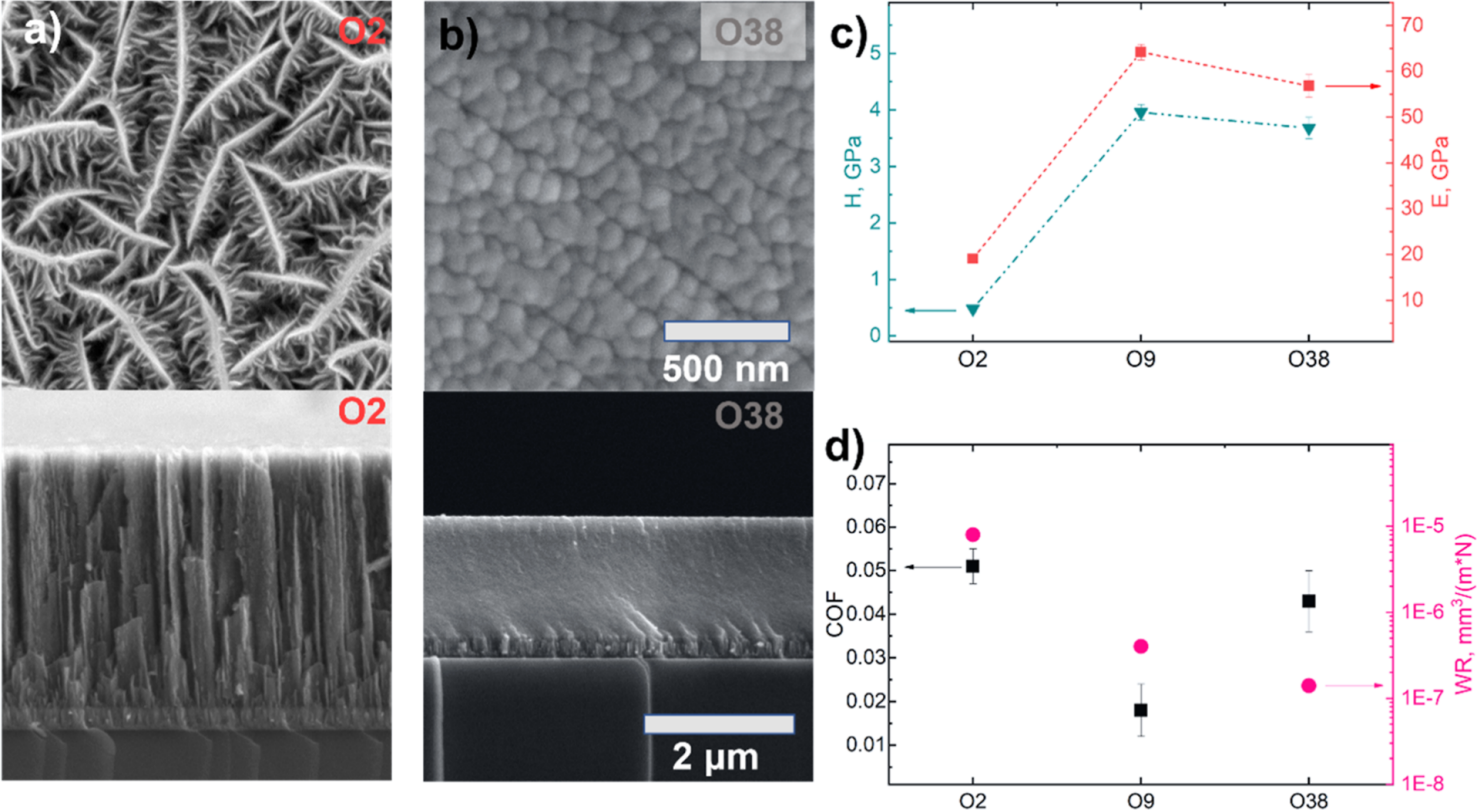
Morphology, microstructure, mechanical, and tribological properties of the coatings. (a) Top-view and cross-sectional SEM images of the O2 and (b) O38 coatings showing the evolution of the microstructure [scale bars are the same for (a,b)], (c) relationships between the chemical composition of the coatings, their hardness, and Young’s modulus, and (d) their steady-state COF and wear rate values.

Figure 2a,b shows the top-view and cross-sectional SEM images of the O2 and O38 coatings. Flake-like topography typical for sputtered TMDs^13,32,70^ turns into an almost featureless surface with some equiaxial elements less than 100 nm in diameter, similar to MoO_*x*_S_*y*_ and MoO_*x*_ films.^46,71^ In the cross section, the columnar morphology disappears when O is incorporated into the MoS_2_ coating (Figure S2). Hardness (*H*) and Young’s modulus (*E*) values of the O2 coating are in good agreement with the existing data for the coatings with the same amount of oxygen and structure^44,47^ (see Figure 2c). The changes of *H* and *E* values with an increase of the O concentration from 2 to 38 at. % can be attributed to several factors: (1) addition of oxygen leads to densification of the structure, represented by a porous columnar structure for O2; (2) for an amorphous material, one can expect better mechanical properties with oxygen addition since the Mo–O bond energy (560 kJ mol^–1^) is higher than the Mo–S bond energy (433 kJ mol^–1^).^72,73^

The COF of O2 is 0.05 and drops to 0.02 and 0.04 for O9 and O38, respectively. Again, the tribological results of the O2 coating are in good agreement with the existing data.^20,30,46^ The O-doped (up to 38 at. %) coatings reveal a distinctively better wear resistance, which decreases with increasing oxygen content. The surface of the wear track of the O2 and O9 coatings is almost featureless, whereas O38 shows shallow scratches aligned parallel to the sliding direction, likely produced by plowing the harder wear debris particles (Figure S3). The images of the counterparts show the tribofilm formation and no wear of the 100Cr6 balls. It is worth noting that the contact area size correlates with the COF value under vacuum (Figure S4). The O9 coating with the lowest friction exhibits the narrowest wear track and the smallest contact spot on the counterpart. Since such a result contradicts traditional reports, a thorough analysis of the sliding interfaces was carried out.

**Figure 3:**
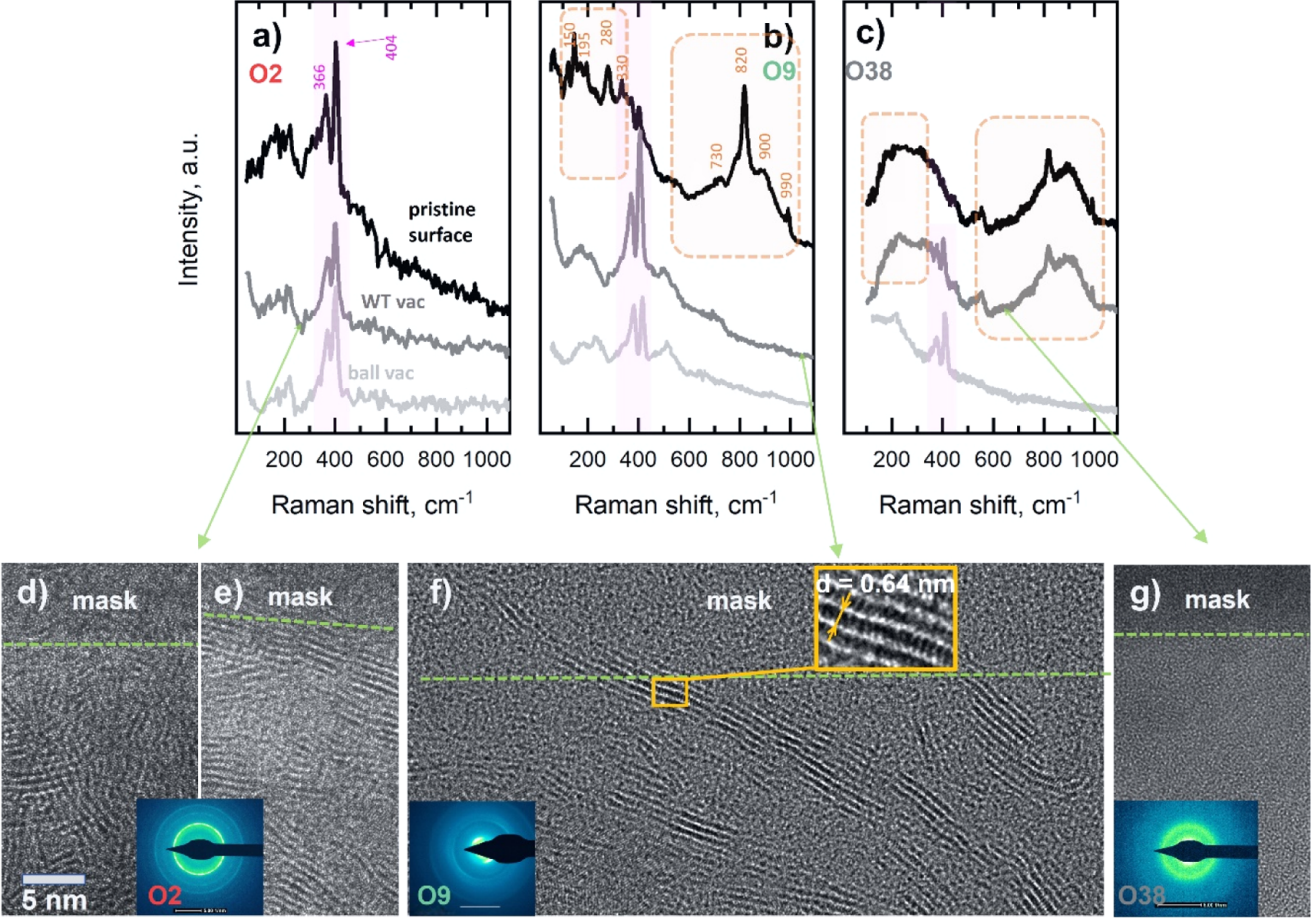
Structure characterizations. Triboactivated phase segregation and formation of a lubricious crystalline component. Raman spectra taken from (a) the pristine O2, (b) O9, and (c) O38 coatings, and spectra taken after their tribological tests from the wear tracks and counterpart surfaces. Cross-sectional HRTEM images of the topmost part of the tribolayers formed on the bottom of the wear tracks on (d,e) O2, (f) O9, and (g) O38 coatings.

Raman spectra from the O2 coating (Figure 3a) reveal two predominant peaks at ≈366 and ≈404 cm^–1^ assigned to MoS_2_ E_2g_ [in-plane vibration in the (100) plane] and A_1g_ [out-of-plane vibration in the (002) direction] first-order Raman vibrational modes, respectively.^74^ Additionally, the asymmetries of the E_2g_ peak and the LA(M) peaks at 150–230 cm^–1^ correspond to the disordered and defected MoS_2_ structure.^75^ These signs of disordered and defected MoS_2_ become less pronounced on the spectra from the ball and the wear track after sliding, suggesting ordering and crystallization of the MoS_2_ in the contact. The spectrum from the as-deposited O9 coating (Figure 3b) exhibits bands centered at 151, 195, 280, 330, 498, 730, 820, and 990 cm^–1^, typical of poorly crystalline/amorphous MoO_*x*_, where 2 < *x* ≤ 3;^76−80^ MoO_*x*_S_*y*_ cannot be excluded either, as it exhibits peaks at 204, 229 and 498 cm^–1^.^81^ Both spectra from the wear track and the counterpart reveal a dramatic change compared to the pristine surface as the MoO_*x*_ peaks vanished and sharp MoS_2_ E_2g_ and A_1g_ bands appeared. Finally, the O38 coating surface shows the broadening of the peaks assigned to MoO_*x*_, suggesting a progressive disordering of the structure; peaks belonging to the MoS_2_ phase are not detected. The spectrum from the wear track of the coatings shows a co-existence of MoO_*x*_ and MoS_2_ bands, although the latter is with a rather weak intensity; moreover, only MoS_2_ bands are visible in the spectra collected from the ball surface (Figure 3c).

A TEM analysis of the O2 wear track cross section identifies a characteristic fringe contrast corresponding to the (002) interplanar spacing of the MoS_2_ phase. The tribolayer is not homogeneous, combining areas of randomly oriented crystallites with areas where basal planes are parallel to the sliding direction (Figure 3e,f). In the case of the O9 coating, the alignment of the MoS_2_ crystallites is more pronounced compared to that of O2. Additionally, the selected area electron diffraction pattern from the wear track area shows only poorly resolved rings corresponding to the h-MoS_2_ phase. The Figure 3g HRTEM inset shows the details of a slightly distorted MoS_2_ crystallite with a (002) interplanar spacing of 0.64 nm. Finally, the surface of the O38 wear track is amorphous. The TEM observation of the wear track surfaces corroborates Raman spectroscopy, but it does not explain the excellent tribological properties of the O38 coating. Since a fully amorphous material is expected to show a much higher COF, we have also employed TEM analysis on the counterpart surface.

**Figure 4:**
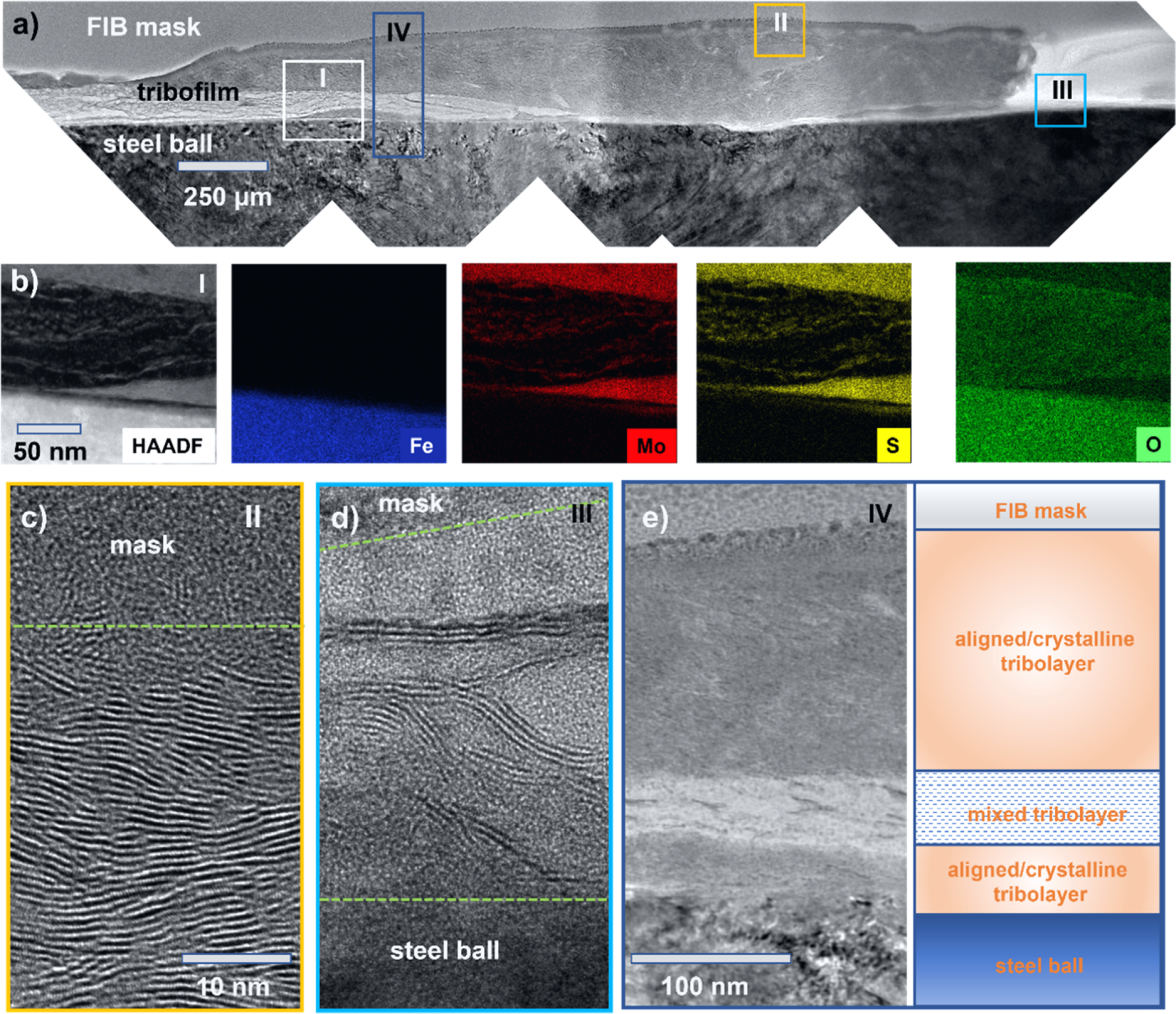
Structure characterizations of the transferred tribolayer. (a) Cross section of the tribolayer on the ball surface with several identified areas for detailed analyses such as (b) chemical mapping or (c,d) HRTEM imaging. Scale bar for (d) is the same as for (c). For area IV, the (e) HRTEM image is combined with a graphical representation of the tribolayer structure.

The tribolayer on the ball is not continuous, consisting of thin areas and thick patches (Figures 4a and S5). The EDX mapping of the cross section suggests that dark gray parts contain oxides, whereas lighter areas are rich in sulfur and molybdenum (Figure 4b). Thicker parts are represented by MoS_2_ with the (002) planes aligned almost parallel to the surface and thus the sliding direction (Figure 4c). The thin parts of the tribolayer show only few-layered elongated MoS_2_ crystallites (Figure 4d), which are also preferentially aligned with the surface. We can summarize here that both Raman spectroscopy and TEM have revealed the triboactivated formation of a MoS_2_ crystalline tribolayer regardless of the oxygen content of the coating. However, for oxygen-doped films, MoS_2_ is always at least partially mixed with amorphous oxide (Figure 4e).

**Figure 5:**
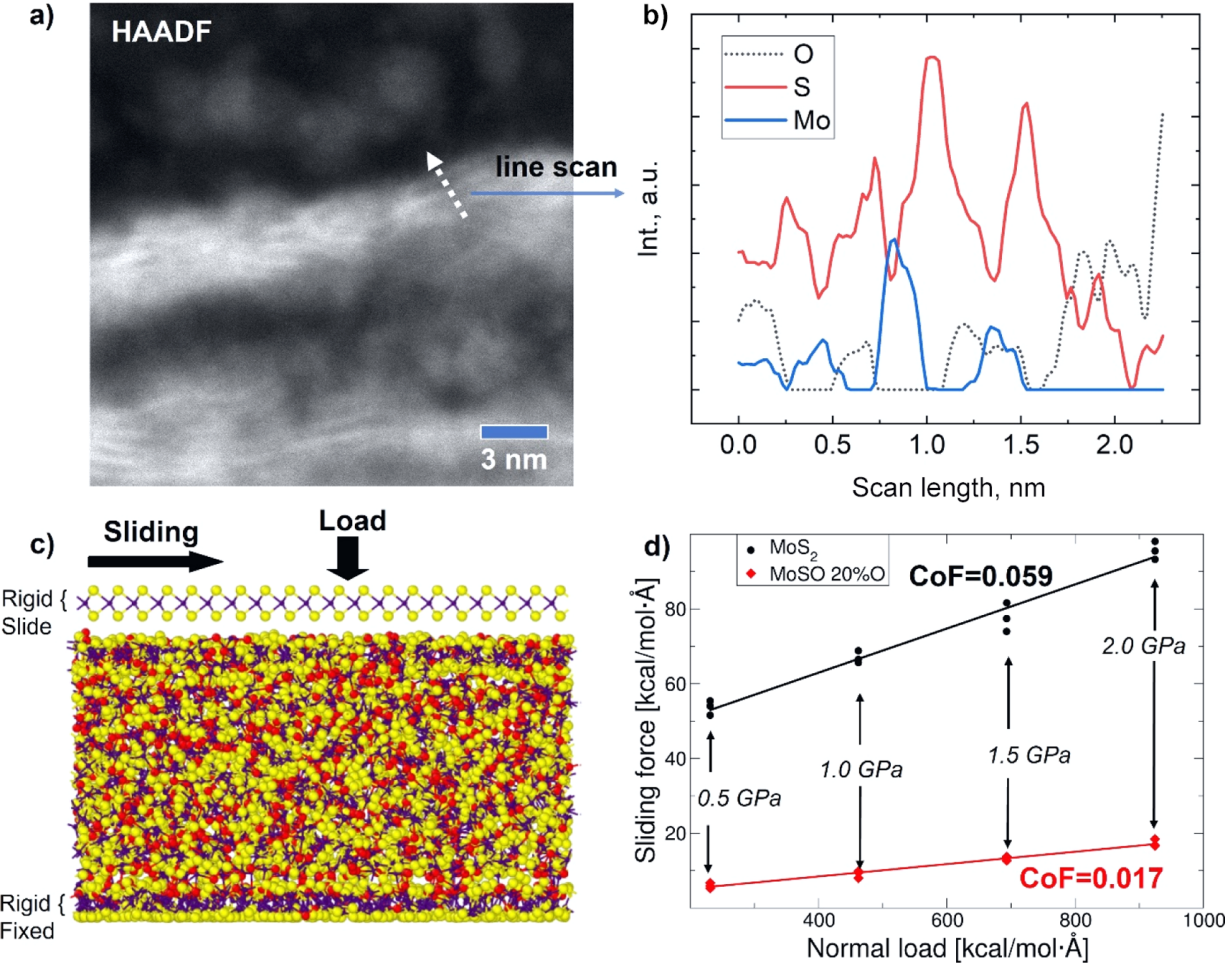
(a) STEM high-angle annular dark-field image of the tribolayer formed on the counterpart surface (coating O38) that consisted of amorphous and crystalline phases, (b) EDS line scan across the crystalline area showing the presence of oxygen in the structure, (c) sliding simulation setup for the Mo–S–O model, and (d) sliding force vs normal load in the sliding simulations for the crystalline MoS_2_ and the amorphous Mo–S–O vs the crystalline MoS_2_.

Why is the COF of the O9 and O38 coatings comparable to (or even lower than) that of the O2 coating? One possibility is that the MoS_2_ formed in the tribolayer is oxygen-free and that the sliding takes place only in this area. We have performed a detailed scanning TEM (STEM) imaging (Figure 5a) and a chemical profile across the MoS_2_-rich part of the tribolayer; there is clear evidence of oxygen embedded within the MoS_2_ layers (Figure 5b). Is it possible that a combination of amorphous Mo–S–O and crystalline pure or O-doped MoS_2_ exhibits a COF similar to that of pure MoS_2_? To answer this question, a series of MD simulations were performed.

To gain more insights into the structure and sliding performance of amorphous Mo–S–O materials, we employed a newly developed Mo–S ReaxFF potential,^82^ which was expanded to include oxygen. The modeled amorphous coating, shown in Figure S7, is a good depiction of the as-deposited amorphous Mo–S–O coating. Even a relatively moderate energy input (short annealing) leads to the onset of the formation of a layered MoS_2_-based phase on the top and the bottom parts of the structure, that is, those regions of the periodic cell that are in contact with the Lennard-Jones walls. The oxygen content in those semi-formed layers is lower than in the overall model: 13 at. % instead of 20 at. %, which is to be expected since the substitution of the S atom into the O atom in MoS_2_ is energetically unfavorable (Figure S8).

Further sliding simulations for the Mo–S–O model were then performed. The top semi-formed MoS_2_ layer was peeled off and replaced with a perfect crystalline MoS_2_ layer, as shown in Figure 5c. This setup mimics the sliding of the formed tribolayers over the remaining amorphous part of the coating. Experimentally, such a structure of the tribofilm was found on the wear track surface (O2, O9) and/or on the counterpart surface (O38). As a point of comparison, sliding in a pure (O-free) MoS_2_ model of the same size was studied. Due to the very strong tendency of MoS_2_ to crystallize and form commensurate layers,^82^ a 2H-MoS_2_ setup was chosen as the starting point. The outcomes of the tribological simulations (in terms of the sliding force vs the normal load) are shown in Figure 5d. The Mo–S–O model shows a notably lower COF (0.017) compared to that of the MoS_2_ (0.059) model, which corresponds well with the experimental part of this study.

Another notable point is the chemical composition of the newly formed tribolayer. It can be seen in Figure S6b that some of the S atomic positions are occupied by the O atoms in the lattice, which was also detected experimentally by atomic-scale EDS profiling. From these results, it can be assumed that even in the case of a partial segregation into amorphous Mo–S–O and crystalline MoS_2_-based phases, low COF values are achievable. When comparing these results with O-free crystalline MoS_2_, it is necessary to mention that relatively deep energy minima on the “sliding map”” (potential energy surface) are characteristic for crystalline 2H-MoS_2_ commensurate (sliding) surfaces.^83^ In the context of our simulations, higher energy barriers to sliding indicate higher COF values. Introducing defects into the 2H-MoS_2_ structure, via partial substitution of S atoms onto O atom positions, can change the potential energy surface significantly. Another possible effect of the presence of O is a slower crystallization (but not stopping completely) so that sliding in a Mo–S–O coating happens primarily on a crystal–amorphous interface free of deep potential energy minima.

Drawing a line under the experimental and computational results, it is worth noting that even the relatively high oxygen content in the film was not an obstacle to the formation of an MoS_2_-rich tribolayer. It seems that the phenomenon governing the tribological properties takes place on the surface of the ball and that the formation of the aligned MoS_2_ on the ball surface is enough to provide lubrication for the system. Meanwhile, the question about the mechanism of the material transfer from the wear track to the ball surface is still open. The exact mechanism probably depends on the amount of Mo and S in the coatings when the as-deposited amorphous material acts as the reservoir from which the crystalline MoS_2_ is delivered to the tribocontact area. In the case of the O2 and O9 coatings, with a relatively high amount of Mo and S, the crystalline MoS_2_ tribolayer was formed on both the wear track and ball surfaces, but the O-doped O9 coating demonstrated the lowest COF (0.02) under vacuum. The lowest COF was accompanied by the smallest tribocontact area (both the narrowest wear track and the smallest contact area on the ball), a smooth and continuous tribolayer inside the wear track consisting of the MoS_2_ and MoS_2_ tribofilm transferred to the ball surface. In the case of the O38 coating, with a lower content of Mo and S, the MoS_2_ tribolayer was mainly formed on the ball surface. We suppose that in the case when the amount of Mo and S is not enough to cover both the wear track surface and the ball surface, MoS_2_ initially goes to the ball surface as soon as it is formed.

As mentioned earlier, triboactivated separation of the Mo–S–O amorphous coatings into a crystalline MoS_2_-based phase and an amorphous S-depleted Mo–S–O balance was found both experimentally and in MD simulations. Despite segregation, no direct evidence of the crystallographic alignment of the tribolayer inside the wear tracks was detected by TEM. Raman spectroscopy showed the formation of the MoS_2_ tribolayer also on the counterpart surface, and the TEM analysis of the tribolayer formed on the counterpart surface after the friction test of the O38 coating under vacuum revealed that its thickness and composition are not homogeneous. The formed tribolayer consists of patchy debris and some thin (<50 nm) continuous film covering the tribocontact area. The structure of the tribofilm also suggests that the friction mode under vacuum for the O38 coating is interfacial sliding between the transfer film and the wear track, together with transfer film shearing. The COF trend agrees with the size of the contact spot on the ball as well as with the amount of the transferred wear debris: a lower COF value comes together with a smaller contact spot and a smaller amount of the transferred wear debris (O9). Perhaps it can be attributed to a change of the friction mode from mixed interfacial sliding/transfer film shearing to interfacial sliding between the transfer film and the wear track and back to interfacial sliding/transfer film shearing in a sequence O2 → O9 → O38.^84^

## 4 Conclusions

In this work, we emphasize the importance of knowing the oxygen concentration in the as-deposited MoS_2_-based coatings. The presence of oxygen can significantly alter the properties of such coatings and can lead to inconsistency between data describing, at first glance, the same material. We demonstrate that, contradictory to common knowledge, adding oxygen to a solid lubricant MoS_2_ coating may be beneficial. Hardness, elastic modulus, wear resistance, and the COF can all be enhanced with the addition of oxygen.

Experiments and MD simulations show that a high oxygen content in the Mo–S–O coatings, up to 38 at. %, does not prevent the formation of a lubricious crystalline MoS_2_ tribolayer, which is formed through triboactivated segregation of the deposited Mo–S–O amorphous coatings into a crystalline MoS_2_-based phase and an amorphous S-depleted Mo–S–O phase. This lubricious MoS_2_-based tribolayer, formed on the surface of the harder Mo–S–O coatings, provides lower COF values compared to an identical tribolayer formed on the surface of softer MoS_2_ coatings (0.02 and 0.05, respectively). Furthermore, MD simulations confirm that sliding of amorphous Mo–S–O against crystalline MoS_2_ results in a COF lower than that of commensurate MoS_2._

The term “oxidation”, which ordinarily signifies a degradation in the tribological performance of MoS_2_, provides only a partial description of the process. In this work, we show that phase segregation governs the tribological behavior, and the presence of chemical bonds with the oxygen itself does not necessarily play a negative role. Since eliminating oxygen in MoS_2_-based solid lubricants is extremely costly, our findings can significantly reduce the production and storage cost for aerospace and other vacuum applications.

## Supporting Information Available

Experimental data (XPS spectra, top-view and cross-sectional SEM images of the as-dep coatings, SEM images of the wear tracks, optical images of the counterparts after tribotests, COF curves vs distance, and SEM images of the counterpart after tribotest) and simulation part (structures in the end of the sliding simulations, the Mo–O–S model before and after annealing, the Convex-Hull diagram, and energies of O-substituted MoS_2_ models with respect to the oxide and sulfide in DFT and ReaxFF) (PDF)
, the Mo-S-O forcefield parameters (TXT)
