# Supplementary material for: Friend or Foe? Revising the Role of Oxygen in the Tribological Performance of Solid Lubricant MoS2
Source: ACS Appl Mater Interfaces. 2022 Dec 5;14(49):55051–61. doi: 10.1021/acsami.2c15706 (PMC9756294; doi:10.1021/acsami.2c15706)
Supplement: Supplementary file 1 — am2c15706_si_001.pdf [file am2c15706_si_001.pdf]

Friend or Foe? Revising the Role of Oxygen in the Tribological Performance of Solid Lubricant MoS_2

Andrey Bondarev[#], Ilia Ponomarev[#], Ruslan Muydinov[†], Tomas Polcar[#]*

[#] Department of Control Engineering, Faculty of Electrical Engineering, Czech Technical University in Prague, Technicka 2, Prague 6, 16627, Czech Republic

[†] Institute of High-Frequency and Semiconductor System Technologies, Technical University Berlin, Einsteinufer 25, 10587 Berlin, Germany

[*] Corresponding author. Email: bondaan2@fel.cvut.cz

Keywords: MoS_2, solid lubricant, tribology, microstructure, ReaxFF

Supplementary Information

Experimental data: S1-S5

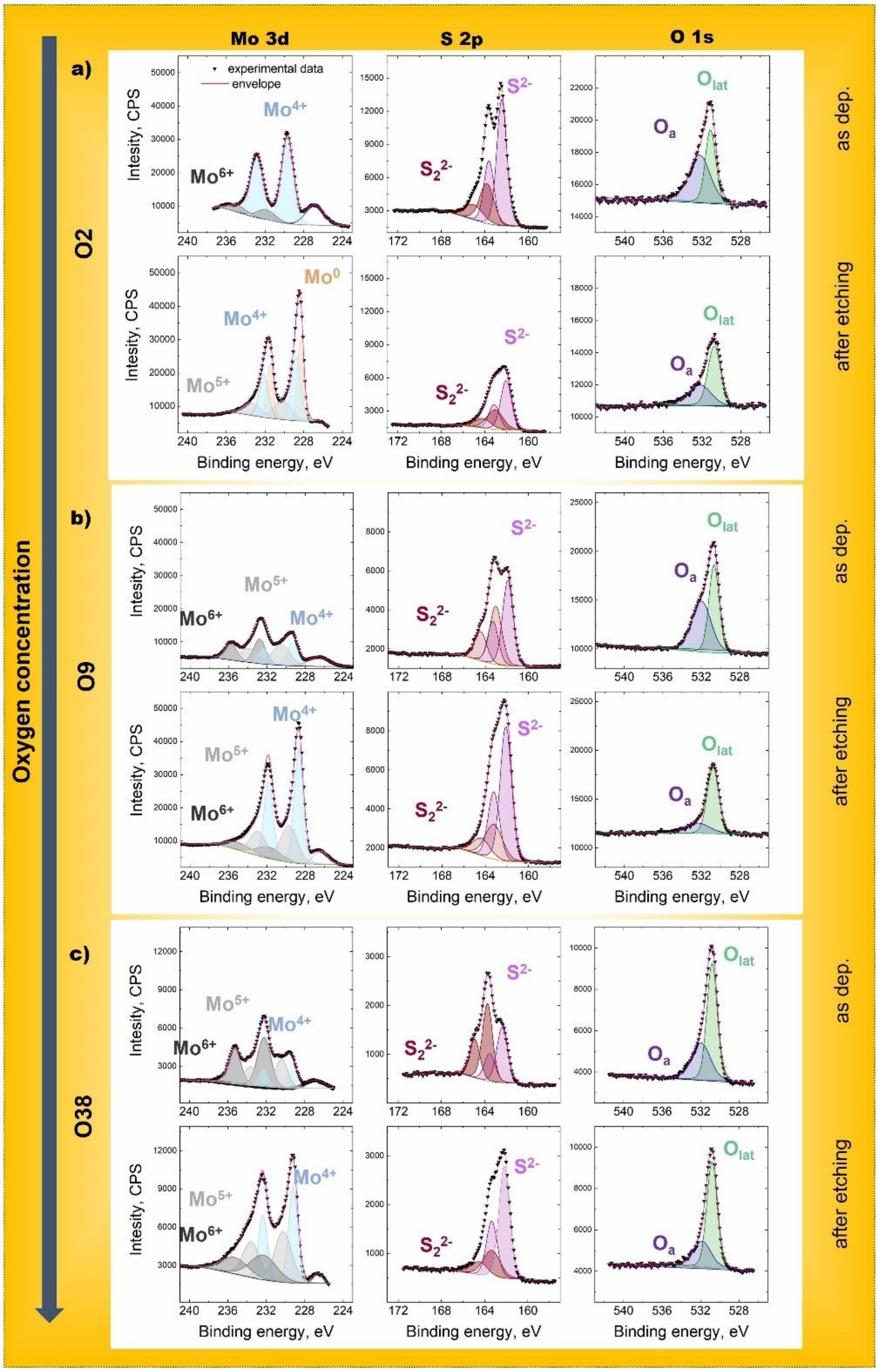

Figure S1. High-resolution Mo 3d, S 2p and O 1s XPS spectra from the surface and after the etching of the coatings (a) O2, (b) O9, (c) O38

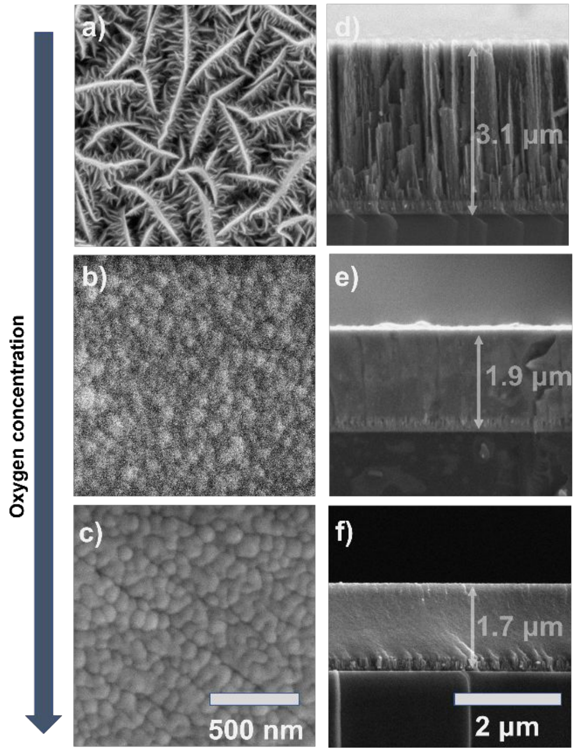

Figure S2. Top-view (a-c) and cross-section (d-f) SE SEM images of the (a,d) O2, (b,e) O9, (c,f), and O38 coatings showing the evolution of the microstructure with oxygen concentration increase

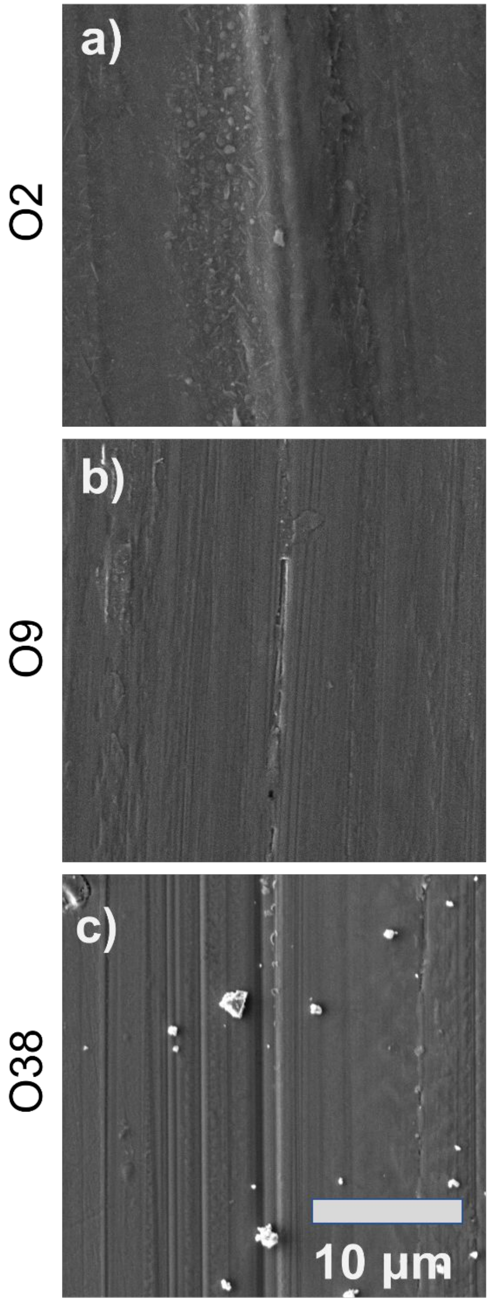

Figure S3. SE SEM images of the wear track bottoms after tribological tests

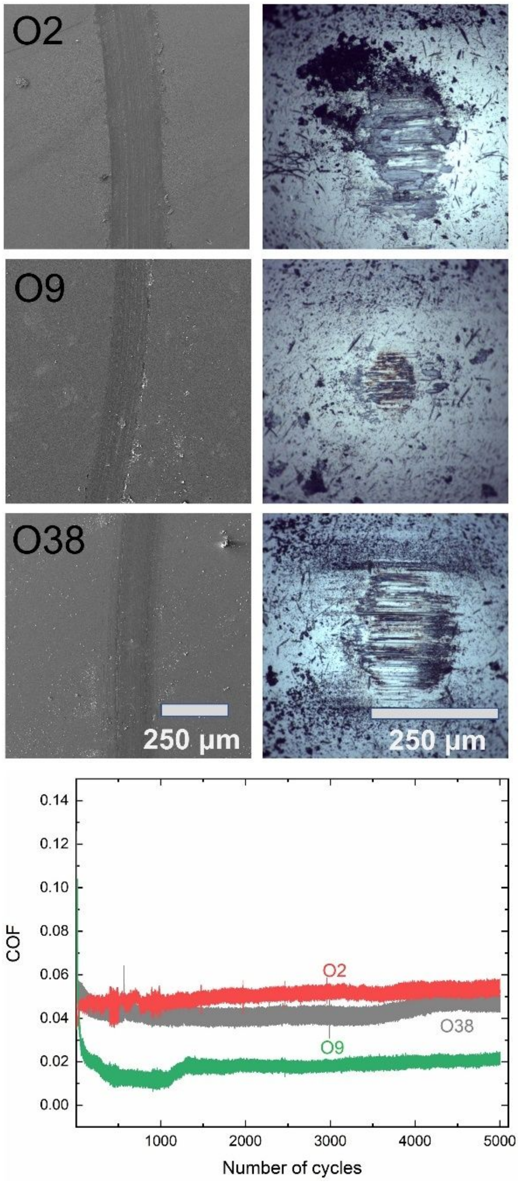

Figure S4. Low magnification SE SEM images of the wear tracks after tribological tests as well as optical images of the counterparts

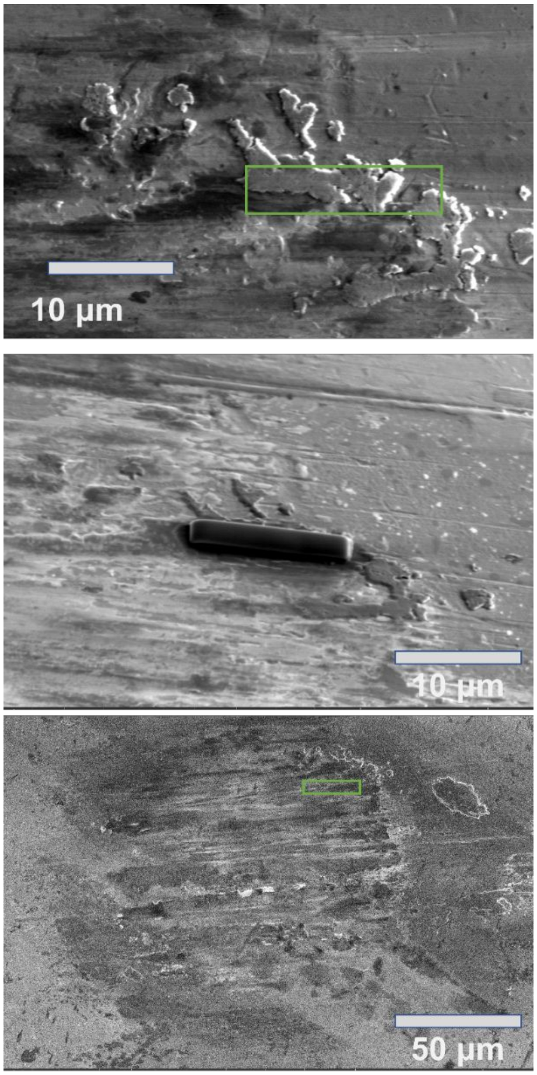

Figure S5. SEM images of the tribolayer formed on the surface of the counterpart after tribological test of the O38 coting under vacuum with a marked place for cross-sectional TEM

Simulation: S6-S9

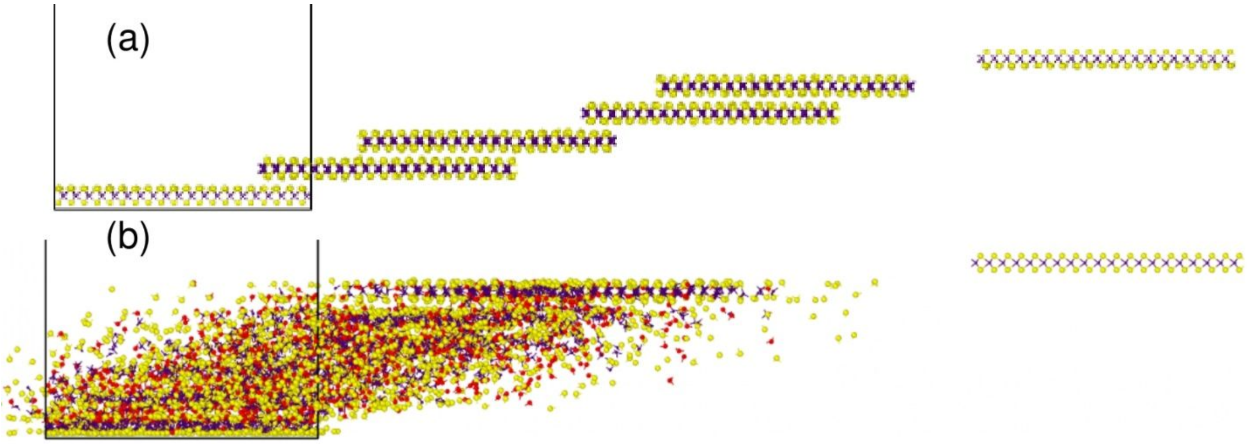

Figure S6. Structures in the end of the sliding simulations (run 1, load 2 GPa, unwrapped coordinates) for commensurate (a) 2H-MoS_2, (b) a-MoSO vs. 2H-MoS_2

Force field development and validation

The development of the Mo-S part of the force field is described in details in the Ref. 61. We used the same reference DFT approach as described in the reference. For the development of Mo-O part of the force field we applied the same Monte-Carlo-like approach and used our Mo-S parameters as the starting point of the Mo-O parameter set development. Figure S8 shows the Convex-Hull diagram of the Mo-O system, comprising experimentally observed MoO_2 (P4/2mnm, P121/c1, Pnma and MoO3 (P121/c1, Pbnm, Pnma structures as well as hypothetical diamond-MoO, rock salt MoO and 2H-MoS_2-type MoO_2. Figure S2 shows the energies (with

respect to 2H-MoS2 and P121/c1-MoO2) of 24-atom MoS2 models with 4 O atoms randomly substituting S atoms.

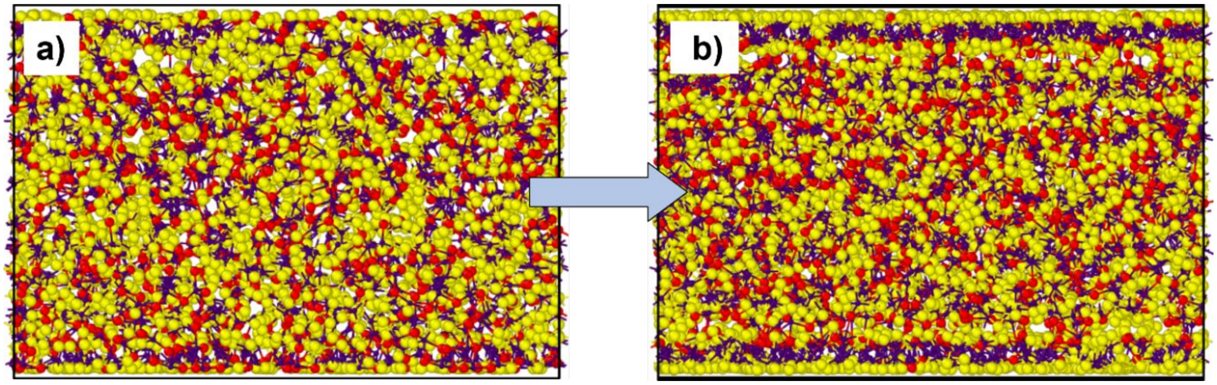

Figure S7. (a) Mo-O-S model in the beginning of the annealing simulation. (b) Mo-O-S model after annealing at 2000 K for 1 ns.

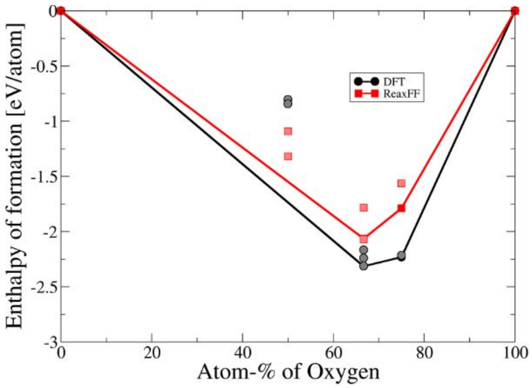

Figure S8. Convex-Hull diagram of the Mo-O system.

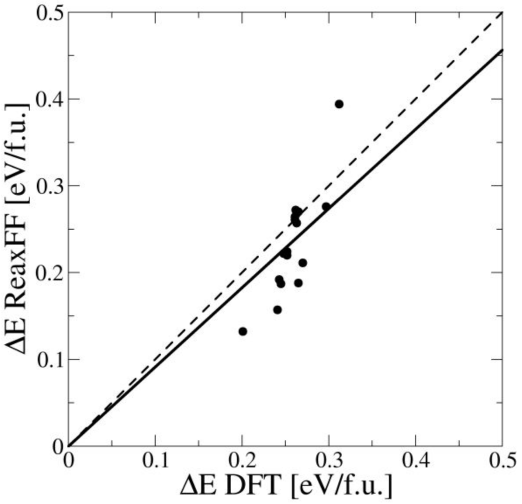

Figure S9. Energies of O-substituted MoS2 models with respect to oxide and sulfide in DFT and ReaxFF. Solid line represents a linear fitting forced through 0. Dotted line is a line with the slope of 1 to guide the eye.

It is not a perfect match of the Convex-Hull diagram, but important features (hypothetical MoO structures are unstable, MoO3 is stable with respect to decomposition into O and MoO2, 2H-MoS2-type MoO2 is notably higher in energy than the most favorable MoO2 structure) are captured. On average substitution of S atoms with O atoms causes about the same changes in energy in ReaxFF as in DFT.
